# Use of four-dimensional computed tomography to aid parathyroid localisation in primary hyperparathyroidism in British surgical practice

**DOI:** 10.1308/rcsann.2025.0062

**Published:** 2025-11-11

**Authors:** N Sawhney, H Findlater, S Hussain, S Rajan, D Bhatt, D McAteer, P Abraham, A Graveling, S Aspinall

**Affiliations:** ^1^ Aberdeen Royal Infirmary, UK; ^2^ Norfolk and Norwich University Hospital, UK

**Keywords:** Primary hyperparathyroidism, Hypercalcaemia or hypercalcemia, Parathyroidectomy, 4DCT, Sestamibi, Effective radiation dose

## Abstract

**Introduction:**

Four-dimensional computed tomography (4DCT) has emerged as an effective imaging modality to aid parathyroid localisation before surgery. Following a service change in 2018, we evaluated the accuracy of 4DCT to lateralise culprit parathyroid glands causing primary hyperparathyroidism (PHPT) in patients undergoing parathyroid surgery in our centre.

**Methods:**

A total of 117 patients underwent 4DCT before parathyroidectomy for PHPT in NHS Grampian between March 2018 and January 2023, of whom 112 underwent first-time operations. Results of imaging, histopathology and surgery type along with biochemical follow-up were evaluated retrospectively to assess the sensitivity and specificity of 4DCT imaging.

**Results:**

In our centre, 4DCT imaging showed results comparable with those reported previously, with 77.6% sensitivity to lateralise to one side of the neck, 89.1% specificity and an overall accuracy of 83.5%. A total of 58% of patients underwent targeted parathyroidectomy. At three- to six-month follow-up, 94.5% had achieved biochemical cure; 4DCT identified culprit lesions in four out of five patients undergoing reoperation. Age and corrected calcium did not affect accuracy of 4DCT.

**Conclusions:**

4DCT is an effective first-line imaging modality before first-time parathyroidectomy for PHPT in patients aged >60 years as well as in cases of reoperation. Use as the first-line imaging modality in younger patients may reduce overall radiation exposure by minimising the need for additional imaging, but further study is needed in this age group.

## Introduction

Primary hyperparathyroidism (PHPT) is caused by the overproduction of parathyroid hormone (PTH) from one or more parathyroid glands, usually resulting in hypercalcaemia. In 80% of cases, a solitary adenoma is found, whereas around 20% are caused by multiglandular disease.^1^ Precise localisation of the culprit gland(s) may enable targeted parathyroidectomy with shortened procedure time, reduced length of hospital stay and complication rates, as well as improved cosmetic results compared with bilateral open neck exploration.^2^

Previous studies reported variable sensitivity and specificity of parathyroid ultrasound (US) and sestamibi-SPECT (sestamibi) in localisation of abnormal parathyroid glands before surgery.^3–7^ In a meta-analysis by Cheung *et al*,^8^ pooled sensitivity for US and sestamibi was 76.1% and 78.9%, respectively. US has the advantage of lower cost and no radiation, but is poorer at identifying ectopic adenomas and is highly operator dependent. Sestamibi helps identify ectopic lesions but sensitivity decreases with smaller lesions and lower PTH levels.^3^ A single-centre, single-surgeon study reported sensitivities of 70% for US, 64% for sestamibi and 81% when combined along with specificities of 57% for US, 57% for sestamibi and 71% when combined.^7^ Concomitant thyroid disease or distorted neck anatomy reduced the sensitivity of these modalities. Radiation from sestamibi increases the risk of colon cancer in all age groups.^9,10^

In the UK, the National Institute for Health and Care Excellence (NICE) (2019) guidance recommends US as first-line and sestamibi as second-line imaging modalities if the results inform a surgical approach.^11^ If the results are discordant or an adenoma is not identified then the surgeon would proceed to surgical four-gland exploration. However, there are now other imaging modalities available to improve localisation before proceeding to surgery.

Four-dimensional computed tomography (CT) (4DCT) involves standard CT imaging during three vascular phases (nonenhanced, arterial and venous) with enhancement over time. Washout of contrast medium with time helps differentiate parathyroid from other structures such as thyroid nodules or lymph nodes, improving the specificity of this test. Mean sensitivity and specificity have been reported as 81.5% and 86%, respectively, in a scoping review including 51 research papers.^12^ 4DCT may be useful in cases of distorted neck anatomy, recurrent parathyroid disease and ectopic adenomas.^13^ However, the utility of 4DCT is limited in younger populations due to a higher radiation dose, particularly to the thyroid gland.^9^

In the North of Scotland we implemented a service change in 2018 to make 4DCT the first-line imaging tool for localising hyperfunctioning adenomas in patients aged >60 years with PHPT or with recurrent disease, and as a second-line option for those aged <60 years who failed localisation with US and sestamibi. We report our experience following this service change by calculating the sensitivity and specificity of 4DCT imaging for parathyroid adenoma, whether this enabled targeted parathyroidectomy and how this affected cure rate in comparison with national standards.

## Methods

### Patient population

A retrospective review of medical records in NHS Grampian of patients aged >18 years who underwent 4DCT imaging before parathyroid surgery for PHPT between March 2018 and January 2023 was undertaken. Some patients underwent 4DCT in addition to sestamibi and/or neck US, if there was uncertainty regarding the location of parathyroid adenoma on the initial scan.

Patients with confirmed hyperparathyroidism were referred to a single endocrine surgeon for consideration of surgery in the presence of symptoms, or if any of the following criteria were present: age <50 years, serum corrected calcium >2.85mmol/l, or if there was skeletal or renal involvement as per guidelines.^1^ Patients who underwent parathyroidectomy for secondary or tertiary hyperparathyroidism were excluded. Patients undergoing first-time and reoperative parathyroid surgery were analysed separately.

Patient demographics, modality and results of imaging, biochemistry before and following surgery, type of surgery undertaken, and excised gland size and weight were recorded. Weight of adenoma was taken from the pathology report. If more than one adenoma was removed from the same side of the neck, the largest was used. Radiology reports were reviewed and correlated with histopathology reports to evaluate accuracy of gland lateralisation.

### Imaging technique

The 4DCT examination was performed using a Siemens SOMATOM X.cite CT scanner, which has 128 detectors and scans at 90kV and 166mAs, with a pitch of 0.8 and slice width of 1mm. An injection of 75ml of iodinated contrast medium (350mg iodine/ml) was infused at a rate of 4ml/s followed by a 25ml saline flush. Image acquisition was performed in three phases: precontrast administration, and at 25s and 80s postcontrast administration. Scan coverage was from the skull base to the carina. The average effective dose in our centre during the study period was 10mSV. All scans were reported by a specialist head and neck radiologist.

### Surgical procedure

A surgeon-performed neck US was undertaken in the operating room to help plan the surgical approach. Parathyroid explorations limited to a single preoperatively localised gland were termed targeted parathyroidectomy. When a search was made for a second gland on the same side of the neck, the operation was termed unilateral neck exploration. The decision to undertake targeted parathyroidectomy or unilateral neck exploration was made intraoperatively at the discretion of the operating surgeon. Bilateral parathyroid exploration was undertaken in several scenarios; preoperative imaging had failed to identify a causative adenoma, known multigland disease, discordant imaging results, or no adenoma found on unilateral exploration. Intraoperative PTH measurement was not available. Serum-corrected calcium and PTH was measured on day one postoperatively and at three- to six-month follow-up.

### Outcomes and analyses

Findings at 4DCT and histopathology were compared to determine the accuracy of 4DCT. This was undertaken as follows: for each side of the neck 4DCT and histopathology reports were categorised as either ‘positive’ or ‘negative’. 4DCT reports noting ‘possible’, ‘probable’ or ‘definite’ parathyroid adenoma were categorised as positive, and reports stating ‘no parathyroid adenoma’, ‘no pathology’ or where no comments were recorded were categorised as negative.

Histopathology reports were categorised as positive where they reported parathyroid adenoma, hyperplasia or carcinoma. Histopathology was categorised as negative when tissue of any other description (e.g. normal parathyroid gland, lymph node, thyroid nodule) or when no tissue was removed at operation. The radiological and histopathological results were then correlated for each side of the neck in every patient. If 4DCT lateralised a pathological parathyroid gland correctly to one side of the neck this was considered successful ‘lateralisation’. Results were then collated to calculate the accuracy of 4DCT.

Cure was determined by normal PTH (reference range 1.6–7.2pmpl/l) and corrected calcium (2.20–2.60mmol/l) at postoperative follow-up. Patients whose corrected calcium or PTH remained elevated were deemed to have persistent disease and those whose corrected calcium fell to the normal range at short-term follow-up but rose to supranormal levels at long-term follow-up were considered as recurrent hyperparathyroidism.

Mean preoperative, postoperative and follow-up corrected calcium and PTH were compared using paired sample *t*-test. Univariable analysis using independent samples *t*-test for continuous variable and chi-square test for categorical variables was undertaken. Pearson’s correlation was calculated for all covariables and those that were correlated strongly and independently (Pearson’s correlation coefficient >0.5) were not both included in the multivariable analysis. Binary logistic regression was undertaken to establish which factors affected accuracy of parathyroid gland lateralisation with 4DCT in a multivariable analysis. Statistical analysis was undertaken with SPSS version 29 software.

## Results

### Patients

A total of 117 patients underwent 4DCT imaging before parathyroidectomy for PHPT in the study period, 5 of whom were undergoing reoperation. The mean age of those undergoing first-time surgery was 69.6 years old (range 35–88) and 86% (96/112) of patients were female (Table 1); 14 patients were <60 years old

**Table 1:** Demographics and baseline imaging and biochemistry results of patients who underwent 4DCT scanning before first parathyroidectomy

Parameter	Value
Number with PHPT who underwent 4DCT	112
Mean age, years (range)	69.6 (35–88)
Sex	86% (96/112) female
CT mean tumour size, mm (range)^†^	19.57 (5–200)
Mean pathological weight, g (range)^‡^	1.94 (0.07–46)
Mean preoperative serum corrected Ca, mmol/l (reference range 2.2–2.6)	2.87 (range 2.47–3.48)
Mean preoperative serum PTH, pmol/l (reference range 1.6–7.2)	27.0 (range 7.1–268.5)

CT = computed tomography; 4DCT = four-dimensional CT; PHPT = primary hyperparathyroidism; PTH = parathyroid hormone

^†^Measured in 77 scans.

^‡^Available in 109 reports.

### Imaging findings

The results for 4DCT lateralisation are shown in Table 2. 4DCT reported a pathological parathyroid on the right side of the neck in 57 patients, on the left side in 36 patients and on both sides of the neck in 4 patients. Of the 112 first-time cases, 62 underwent only 4DCT, 14 underwent 4DCT and formal neck US in the radiology department, 12 underwent 4DCT and sestamibi and 24 had 4DCT, neck US and sestamibi. In cases where multiple scans were performed, the results of previous imaging were available to the reporting radiologist.

**Table 2:** Sensitivity, specificity, positive predictive value, negative predictive value and accuracy of 4DCT in lateralising a pathological parathyroid gland in 112 patients undergoing first-time parathyroidectomy for PHPT

	Performance %
Sensitivity	77.6 (90/116)
Specificity	89.1 (97/108)
Positive predictive value	89.8 (90/101)
Negative predictive value	79.0 (97/123)
Accuracy	83.5 (187/224)

4DCT = four-dimensional computed tomography; PHPT = primary hyperparathyroidism

### Surgical outcome

Of the 112 cases who underwent first-time parathyroidectomy, 65 (58%) had targeted parathyroidectomy (1 combined with hemithyroidectomy and 1 requiring sternotomy for ectopic mediastinal gland) and 7 (6.3%) underwent unilateral neck exploration. The remaining 40 (35.7%) cases underwent bilateral neck exploration (1 combined with hemithyroidectomy and 2 converted from targeted operations). The majority, 81 cases (72.3%), had one parathyroid gland removed at surgery, 22 (19.6%) had two glands and 6 (5.4%) had either three or three and a half glands excised. Three patients had one gland excised and a second gland biopsied.

### Pathology findings

Parathyroid adenoma was diagnosed on histopathology in 92 cases and hyperplasia in 8 cases. In five patients the histopathology was uncertain and in three a thyroid nodule or lymph node was excised. Four patients had parathyroid carcinoma on histopathology, all of whom had normalisation of corrected calcium and PTH postoperatively and at surgical follow-up. Pathological gland weight was recorded in all reports, except for the three where no parathyroid tissue was identified (Table 1).

### Biochemical follow-up

**Figure 1:**
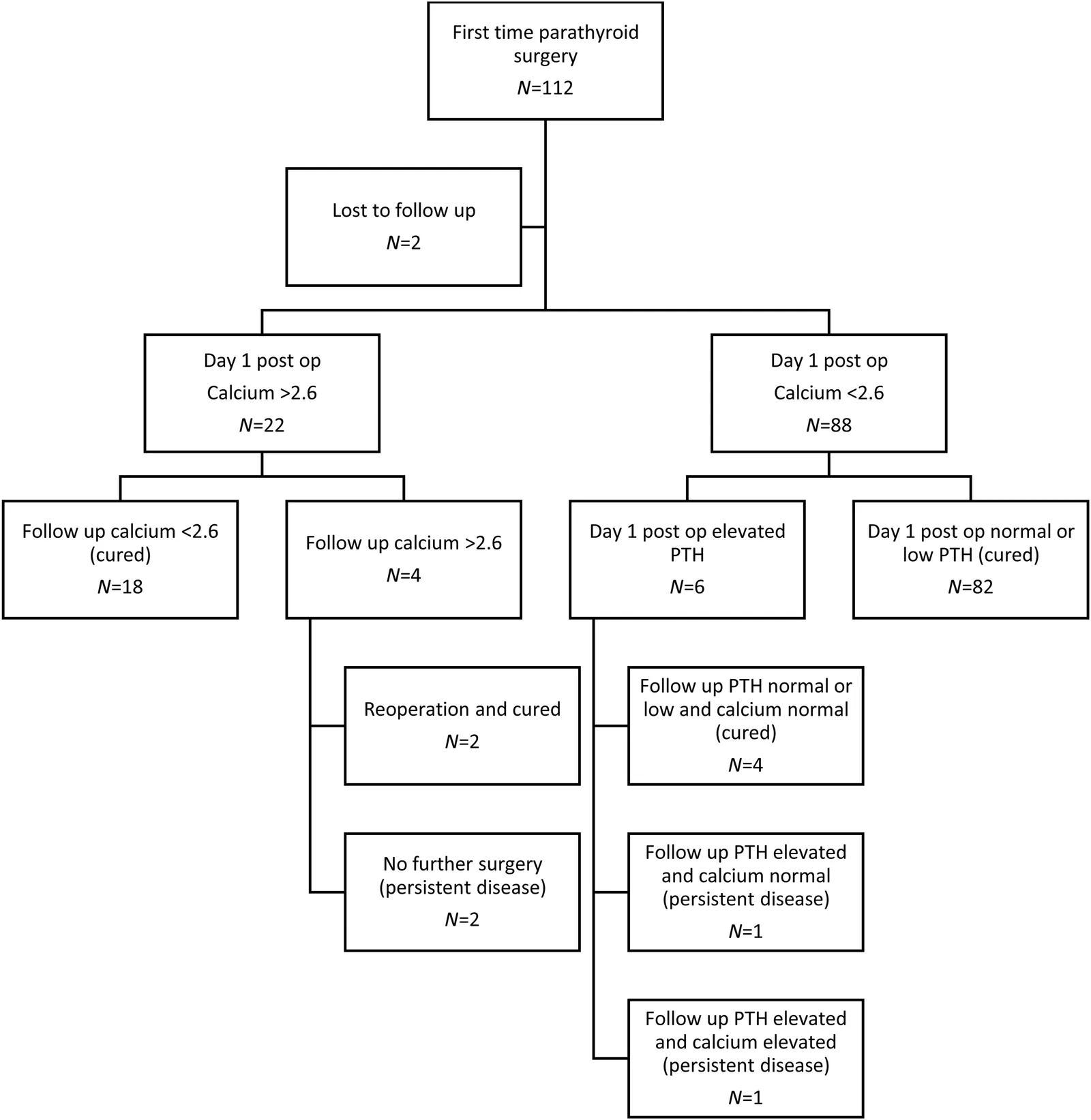
Flowchart showing outcomes from 112 patients who underwent first-time parathyroidectomy for PHPT (median surgical follow-up was 65 days postoperatively (IQR 50–102.5) and long-term follow-up for those cases with persistent disease was 31 months postoperatively (IQR 18.5–44). IQR = interquartile range; PHPT = primary hyperparathyroidism; PTH = parathyroid hormone

Biochemical follow-up was available for 110 cases, of whom 104 achieved cure (94.5%). Of the six not cured, five had persistently elevated serum corrected calcium and PTH with the remaining case having only a raised PTH. Two of these subsequently underwent successful reoperative surgery with normalisation of biochemical parameters, and the remaining four had persistent disease at long-term follow-up (Figure 1).

### Statistical analyses

Preoperative serum PTH and weight of pathological gland were strongly, independently correlated (Pearson’s correlation coefficient 0.669) (Table 3). On univariable analysis there was a trend towards better 4DCT localisation with higher preoperative serum PTH (*p*=0.061, 95% confidence interval (CI) −3.30 to 27.41) and weight of pathological parathyroid gland (*p*=0.078, 95% CI −4.16 to 0.67), but neither achieved statistical significance (Table 3).

**Table 3:** Univariable analysis (independent samples *t*-test) of influence of patient age, preoperative corrected calcium and PTH levels and weight of gland on accuracy of 4DCT parathyroid localisation

	Mean 4DCT correct	Mean 4DCT incorrect	Mean difference	95% CI	*p* value
Patient age, years	69.32	70.71	–1.39	−5.82 to 3.04	0.286
Preoperative corrected Ca, mmol/l	2.88	2.84	0.04	−0.05 to 0.13	0.186
Preoperative serum PTH, pmol/l	29.6	17.6	12.0	−3.30 to 2.74	0.061
Weight of pathological parathyroid gland, gram	2.27	0.53	1.74	−0.67 to 4.74	0.078

CI = confidence interval; 4DCT = four-dimensional computed tomography; PTH = parathyroid hormone

Diagnosis of adenoma on histopathology (74/88 of correct 4DCT compared with 14/24 incorrect 4DCT) was significantly associated with accuracy of 4DCT (Pearson’s chi-square *p*=0.006). Neither patient age (*p*=0.286, 95% CI −5.82 to 3.04) nor preoperative corrected calcium (*p*=0.186, 95% CI −0.049 to 0.131) significantly affected the accuracy of 4DCT (Table 3). Similar results were found on multivariable analysis (Table 4). Of the five patients who underwent reoperative parathyroidectomy, lesions were identified in four on 4DCT and subsequently removed with normalisation of corrected calcium levels in all patients; however, two of these had persistently raised PTH levels at follow-up.

**Table 4:** Multivariable analysis of patient age, preoperative corrected calcium and PTH levels, weight of pathological parathyroid gland and histopathological diagnosis of parathyroid adenoma on accuracy of 4DCT

	Odds ratio	*p* value	95% CI
Patient age, years	0.988	0.647	0.936–1.042
Preoperative serum corrected calcium, mmol/l	1.185	0.910	0.062–22.595
Preoperative serum PTH, pmol/l	1.047	0.096	0.992–1.105
Adenoma on histopathology	2.904	0.066	0.931–9.062

CI = confidence interval; 4DCT = four-dimensional computed tomography; PTH = parathyroid hormone

## Discussion

### Performance of 4DCT

In the present study, the use of 4DCT in lateralising a pathological parathyroid gland in patients undergoing first-time parathyroid surgery for PHPT has been described. It shows this to be an accurate imaging modality for parathyroid lateralisation, enabling over half of cases to undergo targeted parathyroidectomy and facilitating a 94.5% cure rate overall, which is comparable with the national benchmark.^14^

The overall accuracy of 4DCT for lateralising a pathological parathyroid gland to one side of the neck reported in this study was 83.5%: the sensitivity of 77.6% is comparable with that of US and sestamibi combined.^8^ A scoping review by Raemaeckers *et al*,^12^ which reviewed 51 relevant studies, found a slightly better mean sensitivity of 81.5%, similar to the sensitivity of 81% found by Kluijfhout *et al* in their meta-analyses including 34 papers.^15^ At 89.1%, the specificity in our study was slightly higher compared with a mean specificity of 86% in the scoping review by Raemaeckers *et al.*^12^ Differences such as number of cases, CT protocol and previous neck surgery may account for this variance.

The anatomical site of the pathological parathyroid gland was determined in most cases with 4DCT. The ability of 4DCT to lateralise the pathological parathyroid gland as reported in this study suggests that it could be used as the sole imaging modality in PHPT before surgery, as has been adopted in other centres.^16^ As this was a retrospective study and almost half of the patients underwent other imaging modalities, it is difficult to determine how this additional information might have influenced reporting of 4DCT. However, there is an argument for using thyroid US routinely in addition to 4DCT or sestamibi before surgery as US can identify incidental thyroid malignancies that are better dealt with at time of the parathyroidectomy. Given that small, incidentally found, thyroid cancers are often indolent in nature and not therefore clinically significant, there is some uncertainty as to whether identifying and removing them is beneficial.

4DCT was successful at identifying probable adenomas in four out of five patients requiring reoperative parathyroidectomy. This finding is in keeping with previous studies assessing 4DCT accuracy in reoperation. Hamidi *et al* reported that 4DCT was superior to US or sestamibi at identifying culprit lesions in 58 patients with recurrent or persistent PHPT,^13^ with a sensitivity of 77.4% compared with 38.5 and 46% for US and sestamibi, respectively. However, as PTH remained elevated in two of the cases in our study this suggests that not all lesions were identified. Both these patients had parathyroid hyperplasia on histopathology. One had previously undergone a total thyroidectomy and had two enlarged hyperplastic parathyroid glands removed at unilateral neck exploration; a decision was made not to explore the contralateral neck at operation due to extent of adhesions. The other was on long-term lithium and had previously undergone a targeted parathyroidectomy. They underwent reoperative surgery for an adenoma localised to the contralateral side of the neck. Multigland disease can be especially difficult to identify on imaging; however, 4DCT has been shown previously to be superior to sestamibi, with a sensitivity of 53% versus 24%.^17^

### Radiation considerations

The reported effective radiation dose administered to the thyroid gland with 4DCT is variable and dependent on several factors in the CT protocol and patient bodyweight.^12^ Lifetime attributable risks of thyroid cancer from 4DCT scanning and colon cancer from sestamibi scans have been calculated previously.^9,10,18^ Risk of developing thyroid cancer in the over 60-year-old age group following exposure to ionising radiation is negligible, whereas there is increased risk with younger age and female sex. Risk of colon cancer from sestamibi scans, however, remains elevated in all age groups, and thus 4DCT is arguably safer in the older age groups. 4DCT was limited in the <60-year-old age group in our centre to cases where localisation proved difficult with US and/or sestamibi.

The NICE guideline^11^ for parathyroid surgery advises against more than one imaging modality using ionising radiation in first-time parathyroidectomy (published in the second year of patients analysed in this study). Of the 14 first-time parathyroidectomy patients <60 years old in this study, 8 (57.1%) also underwent sestamibi, whereas only 28 out of 98 patients (28.5%) >60 years old underwent sestamibi. This difference is due to an attempt to avoid 4DCT in those <60 years; however, 4DCT has ultimately been required for localisation, resulting in a higher cumulative radiation dose in the younger age group. Stricter adherence to NICE guidelines and development of a departmental protocol for parathyroid localisation may reduce the number of unnecessary scans done in future in NHS Grampian. Further study to identify characteristics of patients less likely to localise with sestamibi and US would help to select those who would benefit from 4DCT as the first-line modality in all age groups. In addition, a re-evaluation of the effective radiation dose with newer 4DCT imaging techniques is required to establish whether sestamibi is still deemed the safer option.

### Study limitations

There were several limitations in our study design. It is a retrospective case series and thus subject to the limitations of such studies. The indications for 4DCT (i.e. was it the initial investigation or was it performed after inconclusive results from other imaging) were difficult to establish retrospectively. The influence of other imaging modalities (i.e. neck US and sestamibi) on overall clinical outcomes are difficult to determine. All patients underwent surgeon-performed neck US in theatre, which may have influenced the outcomes. Surgeon-performed neck US has been shown to aid parathyroid localisation and surgical parathyroidectomy.^19^

Parathyroid lateralisation was chosen as the dependent variable to indicate successful imaging, as the 4DCT radiological reports did not always specify in which quadrant of the neck the enlarged gland was situated. Studies reporting lateralisation of glands in general report higher sensitivities and specificities than those that report localisation of the gland to a quadrant of the neck.

When reporting lateralisation, it is possible that the gland reported on imaging was not the one excised at surgery, or that two enlarged glands on the same side of the neck are found at operation when only one is reported on 4DCT. Thus, there is some uncertainty in this study as to whether the lesion excised at surgery was that identified on 4DCT, which may have influenced the results. However, in the majority of cases, the operative findings corresponded to the 4DCT findings and the small number where this was not the case should not have had a major impact on the overall findings.

Although intraoperative PTH was unavailable in our centre, and it was therefore not possible to confirm operative success during surgery, PTH was measured on the first postoperative day, thus establishing that the causative gland(s) had been excised at surgery. If intraoperative PTH had been available, the outcome of this study could have been affected, as failure of the PTH to fall during surgery in the six cases that developed persistent disease would have prompted further search for pathological parathyroid glands not identified on preoperative imaging.

Finally, the number of cases was too few to show statistical significance when analysing the influence of factors such as preoperative serum PTH and histopathology on the accuracy of 4DCT parathyroid lateralisation. Whereas preoperative calcium levels did not affect the results significantly, calcium levels can be affected by several variables and not only by parathyroid activity. It is interesting to note that age did not affect the accuracy of 4DCT significantly; however, a study including a larger population <60 years would be needed to confirm accuracy in this age group.

## Conclusion

We have demonstrated that 4DCT could be used as a first-line imaging modality before parathyroid surgery for PHPT among people aged >60 years. In addition, it appears to have utility in cases with previous discordant or negative parathyroid imaging. Overall case-mix, diagnostic accuracy and proportions of people subsequently cured were comparable with results reported previously. Further study to identify characteristics of patients less likely to localise with sestamibi and US would help select those who would benefit from 4DCT as the first-line imaging modality in younger age groups to minimise overall radiation exposure.

## Funding

This work was supported by NHS Grampian Charity (grant number GCA25187).

## References

[1] Bilezikian JP, Bandeira L, Khan A *et al.* Hyperparathyroidism. *Lancet* 2018; **391**: 168–178. 28923463 10.1016/S0140-6736(17)31430-7

[2] Laird AM, Libutti SK. Minimally invasive parathyroidectomy versus bilateral neck exploration for primary hyperparathyroidism. *Surg Oncol Clin N Am* 2016; **25**: 103–118. 26610777 10.1016/j.soc.2015.08.012

[3] Zhu M, He Y, Liu T *et al.* Factors that affect the sensitivity of imaging modalities in primary hyperparathyroidism. *Int J Endocrinol* 2021; **2021**: 3108395. 34840566 10.1155/2021/3108395 PMC8616673

[4] Sukan A, Reyhan M, Aydin M *et al.* Preoperative evaluation of hyperparathyroidism: the role of dual-phase parathyroid scintigraphy and ultrasound imaging. *Ann Nucl Med* 2008; **22**: 123–131. 18311537 10.1007/s12149-007-0086-z

[5] Starker LF, Mahajan A, Björklund P *et al.* 4D parathyroid CT as the initial localization study for patients with de novo primary hyperparathyroidism. *Ann Surg Oncol* 2011; **18**: 1723–1728. 21184187 10.1245/s10434-010-1507-0

[6] Rodgers SE, Hunter GJ, Hamberg LM *et al.* Improved preoperative planning for directed parathyroidectomy with 4-dimensional computed tomography. *Surgery* 2006; **140**: 932–941. 17188140 10.1016/j.surg.2006.07.028

[7] Scattergood S, Marsden M, Kyrimi E *et al.* Combined ultrasound and sestamibi scintigraphy provides accurate preoperative localisation for patients with primary hyperparathyroidism. *Ann R Coll Surg Engl* 2019; **101**: 97–102. 30286659 10.1308/rcsann.2018.0158 PMC6351877

[8] Cheung K, Wang TS, Farrokhyar F *et al.* A meta-analysis of preoperative localization techniques for patients with primary hyperparathyroidism. *Ann Surg Oncol* 2012; **19**: 577–583. 21710322 10.1245/s10434-011-1870-5

[9] Mahajan A, Starker LF, Ghita M *et al.* Parathyroid four-dimensional computed tomography: evaluation of radiation dose exposure during preoperative localization of parathyroid tumors in primary hyperparathyroidism. *World J Surg* 2012; **36**: 1335–1339. 22146947 10.1007/s00268-011-1365-3

[10] Hoang JK, Reiman RE, Nguyen GB *et al.* Lifetime attributable risk of cancer from radiation exposure during parathyroid imaging: comparison of 4D CT and parathyroid scintigraphy. *AJR Am J Roentgenol* 2015; **204**: W579–W585. 25905965 10.2214/AJR.14.13278

[11] National Institute for Health and Care Excellence. Hyperparathyroidism (primary): diagnosis, assessment and initial management [NG132]. Issued May 2019. https://www.nice.org.uk/guidance/NG132 (cited July 2025). 31194309

[12] Raeymaeckers S, Tosi M, De Mey J. 4DCT scanning technique for primary hyperparathyroidism: a scoping review. *Radiol Res Pract* 2021; **2021**: 6614406. 34094599 10.1155/2021/6614406 PMC8163538

[13] Hamidi M, Sullivan M, Hunter G *et al.* 4D-CT is superior to ultrasound and sestamibi for localizing recurrent parathyroid disease. *Ann Surg Oncol* 2018; **25**: 1403–1409. 29484563 10.1245/s10434-018-6367-z

[14] Aspinall S, Mihai R, Kinsman R *et al.* Sixth National Audit Report, British Association of Endocrine and Thyroid Surgeons 2021. Henley-on-Thames: Dendrite Clinical Systems Ltd; 2021.

[15] Kluijfhout WP, Pasternak JD, Beninato T *et al.* Diagnostic performance of computed tomography for parathyroid adenoma localization; a systematic review and meta-analysis. *Eur J Radiol* 2017; **88**: 117–128. 28189196 10.1016/j.ejrad.2017.01.004

[16] Leong D, Ng K, Boeddinghaus R *et al.* Three-phase four-dimensional computed tomography as a first-line investigation in primary hyperparathyroidism. *ANZ J Surg* 2021; **91**: 1798–1803. 33982332 10.1111/ans.16924

[17] Galvin L, Oldan JD, Bahl M, *et al.* Parathyroid 4D CT and scintigraphy: what factors contribute to missed parathyroid lesions? *Otolaryngol Head Neck Surg* 2016; **154**: 847–853. 26932954 10.1177/0194599816630711

[18] Moosvi SR, Smith S, Hathorn J *et al.* Evaluation of the radiation dose exposure and associated cancer risks in patients having preoperative parathyroid localization. *Ann R Coll Surg Engl* 2017; **99**: 363–368. 10.1308/rcsann.2017.0014 PMC5449695 28462644

[19] Aspinall SR, Nicholson S, Bliss RD *et al.* The impact of surgeon-based ultrasonography for parathyroid disease on a British endocrine surgical practice. *Ann R Coll Surg Engl* 2011; **94**: 17–23. 10.1308/003588412X13171221498389 PMC3954181 22524912

